# Intensity-Dependent Optical Response of 2D LTMDs Suspensions: From Thermal to Electronic Nonlinearities

**DOI:** 10.3390/nano13152267

**Published:** 2023-08-07

**Authors:** Anderson S. L. Gomes, Cecília L. A. V. Campos, Cid B. de Araújo, Melissa Maldonado, Manoel L. da Silva-Neto, Ali M. Jawaid, Robert Busch, Richard A. Vaia

**Affiliations:** 1 Departamento de Física, Universidade Federal of Pernambuco, Recife 50670-901, PE, Brazil; ceciliaveras@gmail.com (C.L.A.V.C.); cid.araujo@ufpe.br (C.B.d.A.); memac17@gmail.com (M.M.); mleonardofisica@gmail.com (M.L.d.S.-N.); 2 Institute of Physics, Pontificia Universidad Católica de Chile, Santiago 7820436, Chile; 3 Materials and Manufacturing Directorate, Air Force Research Laboratories, Wright-Patterson Air Force Base, Dayton, OH 45433, USA; ali.jawaid.ctr@us.af.mil (A.M.J.); robert.busch.6.ctr@us.af.mil (R.B.); richard.vaia@us.af.mil (R.A.V.)

**Keywords:** electronic optical nonlinearity, thermal optical nonlinearity, bidimensional transition metal dichalcogenides

## Abstract

The nonlinear optical (NLO) response of photonic materials plays an important role in the understanding of light–matter interaction as well as pointing out a diversity of photonic and optoelectronic applications. Among the recently studied materials, 2D-LTMDs (bi-dimensional layered transition metal dichalcogenides) have appeared as a beyond-graphene nanomaterial with semiconducting and metallic optical properties. In this article, we review most of our work in studies of the NLO response of a series of 2D-LTMDs nanomaterials in suspension, using six different NLO techniques, namely hyper Rayleigh scattering, Z-scan, photoacoustic Z-scan, optical Kerr gate, and spatial self-phase modulation, besides the Fourier transform nonlinear optics technique, to infer the nonlinear optical response of semiconducting MoS_2_, MoSe_2_, MoTe_2_, WS_2_, semimetallic WTe_2_, ZrTe_2_, and metallic NbS_2_ and NbSe_2_. The nonlinear optical response from a thermal to non-thermal origin was studied, and the nonlinear refraction index and nonlinear absorption coefficient, where present, were measured. Theoretical support was given to explain the origin of the nonlinear responses, which is very dependent on the spectro-temporal regime of the optical source employed in the studies.

## 1. Introduction

Understanding the light–matter interaction under laser excitation has been an important driver for researchers in the field of nonlinear optics and materials science. Beyond the basic science, technological developments led to a broad range of applications of photonic materials, from theragnostic to optical communications. Among the myriad of materials for photonic applications, ultrathin (below 10’s of nm to just a few nm) two-dimensional (2D) lamellar materials have appeared with appealing applications based on the growth, characterization, and management of processing methods, as reviewed in [1,2,3,4]. On the other hand, nonlinear optics (NLO) and nonlinear photonics studies in a wide range of 2D materials have rapidly evolved in the last 5–10 years, as can be seen in the reported reviews in [5,6,7,8,9,10,11].

The NLO basics and techniques to study an optical material response to an incident field are well-revised in articles [12,13] and textbooks [14,15], and they will not be re-emphasized or reproduced here.

A very important aspect of the NLO response of a medium is the understanding of its origin. For that, it is imperative to employ an adequate spectro-temporal regime for the optical source, as well as the morphological and linear optical characteristics of the medium under study. The measurement techniques are also determinant for understanding the physical processes in the NLO response and may indicate the basis for photonics or optoelectronics applications. The most studied NLO responses arise from the second- and third-order nonlinearities, which leads to wave mixing processes as summarized in Table 1. The second-order polarization leads to second harmonic generation (SHG) and sum or difference frequency mixing (SFM or DFM, respectively) and optical rectification (OR). The third-order polarization leads to a myriad of effects, including third harmonic generation (THG), four-wave mixing (FWM), optical Kerr effect (OKE), saturable absorption (SA), two-photon absorption (TPA), and stimulated Raman scattering (SRS). It is also worth reiterating that the third-order susceptibility, *χ*^(3)^, is a complex parameter with real and imaginary parts, Re$\chi^{\left(3\right)}$ and Im$\chi^{\left(3\right)}$. Those two terms can be described (see one of the refs. [12,13,14,15] for details, or a good summary of all terms in the polarization expansion of $\chi^{\left(3\right)}$ in ref. [8]) as being proportional to the intensity-dependent nonlinear refraction (NLR) index and nonlinear absorption (NLA) coefficient, respectively.

Generally speaking, the real part of $\chi^{\left(i\right)}$ is proportional to the refractive index, $n_{i-1}$, while its imaginary part is related to the absorption coefficient, $\alpha_{i-1}$. In the case of centrosymmetric materials, only the first- and third-order susceptibilities are considered (neglecting higher-order odd terms). The third-order refractive index, $n_{2}$, and the third-order absorption coefficient, $\alpha_{2}$, can be written (in SI units) in terms of $\chi^{\left(3\right)}$ as

(1) $$n_{2}=\frac{3\;Re\;\chi^{\left(3\right)}}{4\epsilon_{0}cn_{0}^{2}}$$

and

(2) $$\alpha_{2}=\frac{3\omega \;Im\;\chi^{\left(3\right)}}{2\epsilon_{0}c^{2}n_{0}^{2}}$$

In the review article of ref. [8], as well as in ref. [12], an excellent account of the NLO techniques employed to characterize optical materials is given, and in this review, we shall not be exhaustive and only describe the techniques that we have employed (see Section 2). Furthermore, in a recent publication, Vermeulen and co-workers reported on “post-2000 Nonlinear Optics: Data Tables and Best Practices” [16], where a thorough survey of NLO in a wide variety of materials, including 1D, 2D, and 3D dimensions, from fibers to nanostructures to bulk, is described, with insights into physical pictures, techniques, and the main results.

**Figure 1:**
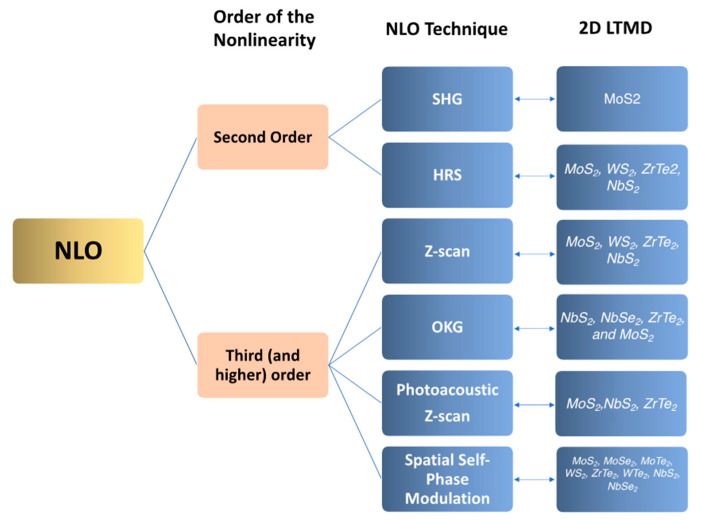
NLO optical processes discussed, techniques employed, and the 2D-LTMDs studied for each technique.

In Figure 1, we pictorially describe what comes in the remainder of this article, which mainly reviews some of our own work over the last few years in characterizing the NLO properties of 2D layered transition metal dichalcogenides (LTMDs) in suspension and comparing it with some reports in the literature. In Section 2, we describe the preparation and characterization of the materials, as well as the employed NLO techniques. In Section 3, we describe the results of intensity-dependent NLO response of 2D LTMDs, particularly second-order studies, intensity-dependent thermal response, and intensity-dependent non-thermal responses, including time-resolved results. In Section 4 we discuss challenges and opportunities for research in 2D LTMDs.

## 2. Material Preparation and Characterization Methods

### 2.1. LTMDs Synthesis and Characterization

**Figure 2:**
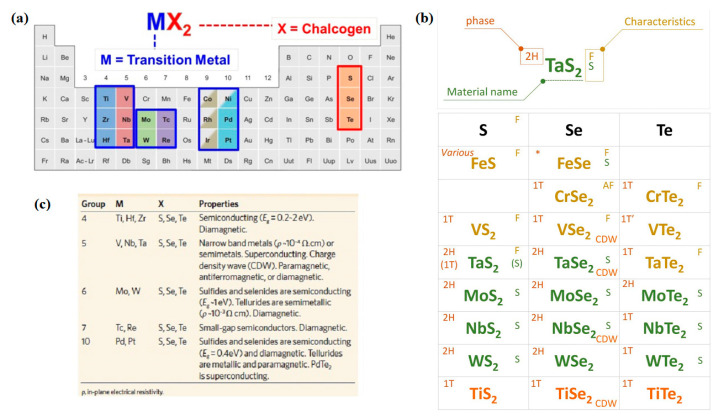
(**a**) Periodical table highlighting the atoms that can be combined to form 2D LTMDs and other 2D materials (from ref. [17], with permission); (**b**) various physical properties of 2D materials (including LTMDs) such as magnetism (ferromagnetic (F)/anti-ferromagnetic (AF)), superconductivity (s) and charge density wave (CDW) and crystal structures (2H, 1T) (adapted from ref. [18], with permission); (**c**) electronic character of different 2D LTMDs (from ref. [19], with permission).

As indicated in refs. [1,2,3,4], there have been great advances in the synthesis of 2D materials, from graphene and beyond, which includes black phosphorous, MXenes, clays, arsenenes, silicenes, phosphorenes, hexagonal boron nitrite, and the LTMDs family, among others. The synthesis methods for such materials vary and follow the general classification of bottom-up or top-down methods. Bottom-up methods, which are excellent for synthesizing high-quality ultrathin nanocomposites with very wide lateral dimensions, include wet chemical, hydro/solvothermal, template, microwave-assisted, topochemical transformation, chemical and/or physical vapor deposition (CVD/PVD). On the other hand, top-down techniques include a variety of exfoliation processes such as electrochemical, ultrasonic, ion exchange, mechanical, and liquid phase exfoliation (LPE). A good review and well-referenced text on the above bottom-up and top-down methods can be found in ref. [3]. Figure 2a shows a periodical table highlighting the atoms that can be combined to form 2D LTMDs and other 2D materials [17], Figure 2b depicts a table exhibiting various physical properties of 2D materials (including LTMDs) such as magnetism (ferromagnetic (F)/anti-ferromagnetic (AF)), superconductivity (s) and charge density wave (CDW) and crystal structures (2H, 1T) [18]. In the table of Figure 2c, other relevant characteristics of 2D LTMDs are shown [19].

As pointed out in ref. [17], TMDs present strong anisotropy in their electrical, chemical, mechanical, and thermal properties, as a consequence of the way they crystallize, in a graphite-like layered structure. TMDs formed from Groups 4–7 (the most studied in this work) in Figure 2a are predominantly layered, whereas some of the TMDs formed from Groups 8–10 are generally found in non-layered structures. It is also known that, in layered structures, the layer typically has a thickness of 6~7 Å, consisting of a hexagonally packed layer of metal atoms sandwiched between two layers of chalcogen atoms. The inner-layer M–X bonds are mainly covalent in nature, whereas the sandwich layers are coupled by weak van der Waals forces, therefore allowing the crystal to readily cleave along the layer surface. Several other fundamental features of 2D LTMDs can be found in the indicated refs. [1,2,3,4,17,18,19].

**Figure 3:**
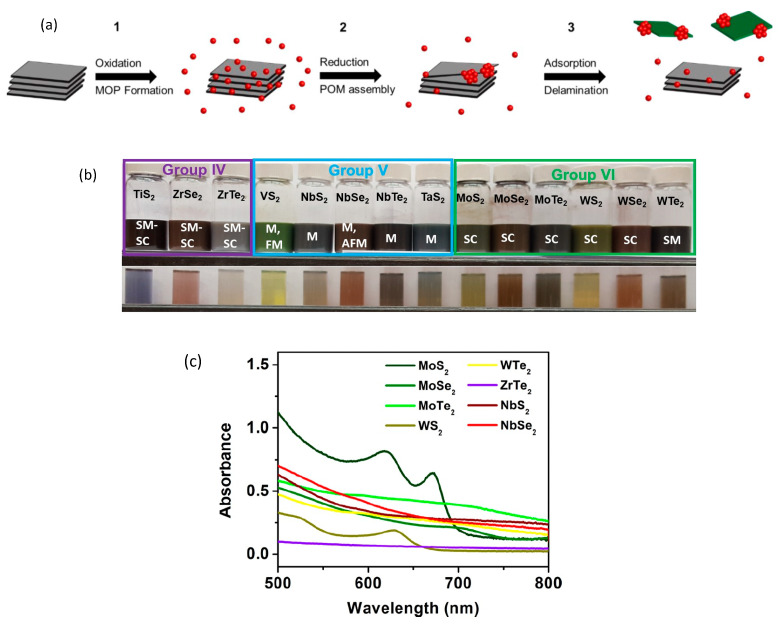
(**a**) Mechanism of redox exfoliation via POM assembly. (1) Bulk MoS_2_ powders are treated with a mild oxidant (cumene hydroperoxide) to generate solution-soluble molecular metal oxide precursors (MOPs described as red spheres) that exist in a solution−surface equilibrium. (2) The addition of reductant initiates MOP condensation and assembly into highly charged polyoxometalates (POMs). These POMs adsorb to MoS_2_ surfaces and create strong Coulombic repulsion driving delamination. (3) Exfoliation of few-layer MoS_2_ from the bulk crystallites exposes fresh surfaces for further adsorption, assembly, and delamination events until precursors (MOPs) and active exfoliant (POMs) are exhausted (from ref. [21], with permission); (**b**) Photographs of the 2D LTMDs colloids prepared using the technique shown in (**a**). SM-SC: semimetal-semiconductor; M-FM: metal-ferromagnetic; M- metal; SC—semiconductor; SM—semimetal (picture from the authors). (**c**) Absorbance spectra for the 2D LTMDs in ACN suspension reported in this review. Further details will be given in the Section 3.

In this review, we focus on an alternative LPE method based on a redox exfoliation mechanism [20,21] to achieve few-to-monolayer yields for MX_2_ (M = Ti, Zr, V, Nb, Mo, and W; X = S, Se, Te), covering Groups 4–7 (see Figure 2a) with the feasibility of exceeding 10% of the starting LTMD powder. The reported selected results employed the nanomaterials produced by this method. All the nanoflakes were suspended in acetonitrile (ACN), providing a stable colloid (see Figure 3). A detailed description of the chemical material acquisition, synthesis, and characterization of the materials is given in refs. [20,21] and only a summarized description is given here, which is illustrated in Figure 3 (from ref. [21]) for groups 4–7 LTMDs. The main steps are: (a) a soluble oxidant is added to a heterogeneous suspension of LTMDs in ACN to form soluble molecular metal oxide precursors (MOPs); (b) following that, a reductant (also soluble) is added to put together these MOPs to anionic polyoxometalates clusters (POMs); (c) adsorption of these POMs to the edge, interlayer gap, or surface of the bulk crystallites triggers sequential delamination via Coulombic repulsion, and provides colloidal stability to the delaminated sheets. It is important to notice that this process does not require mechanical agitation of the LTMDs and stirring (via a stir plate) suffices to start delamination. Furthermore, redox exfoliation appears to be ubiquitous, as soluble metalates (SoMs = soluble MOPs and POMs) are typical products of Groups IV–VII LTMD oxidation via hydrolysis. Therefore, as shown in [20,21], redox exfoliation has been shown to provide stable, colloidal dispersions of all Groups 4–7 LTMDs in a wide range of solvents (e.g., acetone, acetonitrile, and DMF), as seen in the photographs of Figure 3b. Because of batch-to-batch variability and an incomplete understanding of the multistep procedure, its broader utilization has been limited, despite its generality.

Morphological and optical characterization of the 2D LTMDs were carried out using different techniques, which shines a light on the main features of the synthesized nanostructures. Again, a detailed account is given in refs. [20,21], and their support information therein, for all prepared suspensions reviewed in this work. Data showing AFM, TEM, XPS, and Raman spectroscopy results give the relevant information. Another important aspect of the materials employed was their stability. Throughout the different experiments, the material stability was checked visually (for flocculation or decantation) and the ones where it happened (few samples from all the batches) were not used. Most of the samples were and still are stable and stored in the refrigerator when not in use, and all experiments were performed at room temperature for several days or weeks, without any modification of the sample. Also, extinction/absorption spectra were taken before and after using the samples for long (weeks) periods to verify that no modification occurred.

### 2.2. NLO Techniques

**Figure 4:**
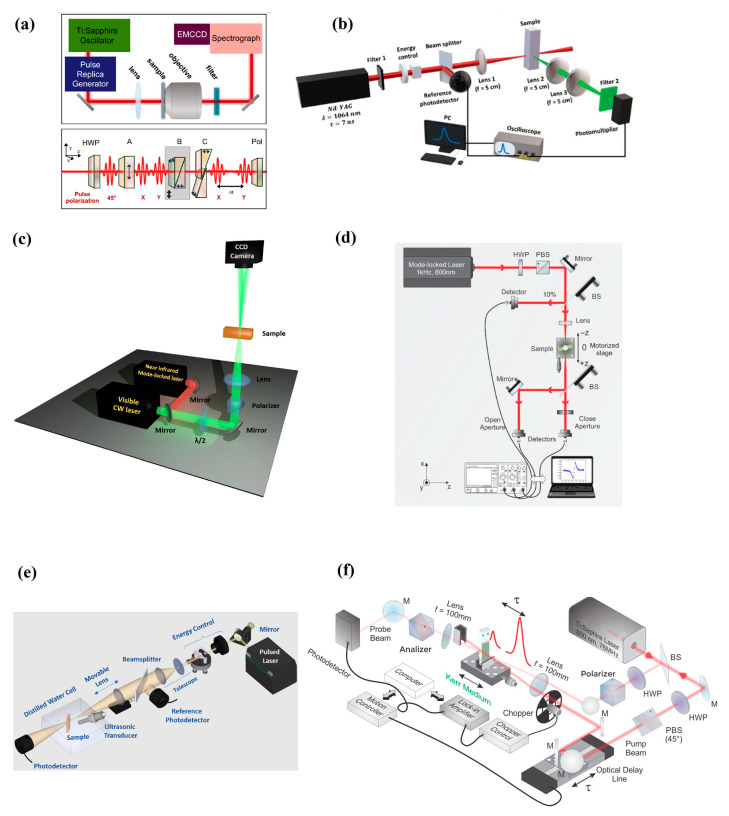
NLO methods employed in the reviewed work. (**a**) Fourier Transform Nonlinear Optics (FT-NLO) [23]. For further details regarding blocks A, B, and C, see Ref. [23]. (**b**) Hyper Rayleigh Scattering (HRS) [25]; (**c**) Spatial Self-Phase Modulation (SSPM); (**d**) Z-scan; (**e**) Photoacoustic Z-Scan (PA Z-scan); and (**f**) Optical Kerr gate (OKG) (Figures reproduced from the indicated references with permission).

NLO techniques have been widely exploited and well-reviewed in the literature for photonic materials characterization. In particular, refs. [8,12,16] bring the relevant aspects of the majority of the NLO methods recently employed, particularly for 2D materials [8]. In the works whose results will be discussed here (refs. [22,23,24,25,26,27,28,29,30]), six different methods were employed, as shown schematically in Figure 4a–f, namely: (a) Fourier Transform Nonlinear Optics (FT-NLO); (b) Hyper Rayleigh Scattering (HRS); (c) Spatial Self-Phase Modulation (SSPM); (d) Z-scan; (e) Photoacoustic Z-Scan (PA Z-scan); and (f) Optical Kerr gate (OKG). A brief description of the basics of all six methods is given. The results will be reviewed and discussed in Section 3.

#### 2.2.1. Fourier Transform Nonlinear Optics (FT-NLO)

Fourier Transform Nonlinear Optical (FT-NLO) spectroscopy is a powerful tool that enables the resolution of high-order effects based on interferometric measurements of the signal [23]. To implement this technique, a pulse replica generator (PRG) is necessary to allow measurements as a function of the delay (Δτ) between the optical pulses. Coherent (phase-locked) pulses can be obtained by employing a birefringent delay line, as described in refs. [31,32], and are schematically represented in Figure 4a (bottom setup).

The FT-NLO technique relies on the Fourier transform (FT) of spectrally and time-resolved interferometric measurements of the NLO signal to retrieve information about high-order effects [23] and NLO effects of very low intensity, as in the case of resonance response of individual gold nanorods [33]. The NLO signals of interest are obtained after focalizing the pulse replicas in the sample by using a converging lens, followed by an objective lens to collect the signal that will further be filtered before being detected for analysis (Figure 4a, top setup).

#### 2.2.2. Hyper Rayleigh Scattering (HRS)

HRS is a technique first employed for studies of molecular solutions [34] and can be used to determine the orientation-averaged hyperpolarizability of molecules by correlating the intensity of the scattered second harmonic (SH) wave with the molecular concentration [35]. A typical experimental setup employed for the HRS measurements is shown in Figure 4b. To determine the first-hyperpolarizability, an external reference method can be conveniently employed using a known material such as para-nitroaniline (p-NA) as the reference standard [36]. The theoretical treatment for the HRS data is well-established [34,35], as is the external reference method [36]. The HRS signal, $I\left(2\omega \right)$ as a function of laser intensity for the two-component (suspension with solute + solvent) system studied, can be written as [37]:

(3) $$I\left(2\omega \right)=G(N_{sol}\langle \beta_{sol}^{2}\rangle {+N_{solv}\langle \beta_{solv}^{2}\rangle )I}^{2}\left(\omega \right),\;$$

where *sol* stands for solute (the nanoflakes), *solv* stands for solvent, and G is a parameter that includes local field correction and light collection efficiency. The determination of $\beta_{c}\left(2\omega \right)$ by using the reference method will be further discussed in Section 3, with examples to be given. Other general details can be found in ref. [37].

#### 2.2.3. Spatial Self-Phase Modulation (SSPM)

Among nonlinear optical processes, self-phase modulation of a laser beam occurs when its intensity is sufficiently high to modify its own properties upon propagation in a nonlinear medium. For spatial self-phase modulation (SSPM), in particular, a nonlinear phase that depends on the spatial intensity profile of the beam is acquired, giving rise to distinct diffraction patterns readily observed in the far field. As a third-order NLO effect, SSPM is described from a nonlinear correction in the refractive index, regardless of the origin of the effect, which can be electronic, thermal, or orientational, to cite a few. Therefore, the examination of the physical mechanism underlying the NLO response becomes crucial to avoid misconceptions concerning the NLO properties of the materials since, for instance, intrinsic electronic properties do not bear a relationship with thermal nonlinearities. This is particularly important in studies of SSPM because, unlike spectral phase modulation, which requires optical pulses with high peak intensities, SSPM can be observed using both pulsed and CW lasers. Therefore, with either CW or mode-locked (ML) high-repetition rate (~MHz) lasers, thermal effects should be considered in the analysis.

Figure 4c is the schematic representation of a typical setup to study SSPM in liquid suspensions of 2D materials. The vertical configuration is considered to avoid distortions in the diffraction patterns due to convection [38]. The spatial self-phase modulation of Gaussian beams, as employed in ref. [26], gives rise to a set of concentric rings in the far-field, from where one can retrieve $n_{2}$ based on the number of rings, the incident intensity, the effective length of the sample ($L_{eff}=\frac{1-e^{-\alpha_{0}L}}{\alpha_{0}},$ where L is the cuvette length, $\alpha_{0}$ is the linear absorption coefficient [26,39]), and the light wavelength [26,40].

Conversely, when using structured light, the nonlinear phase acquired by the optical beam follows its own spatial intensity profile, which leads to distinct diffraction patterns in comparison to the SSPM of Gaussian beams. For optical vortex beams (OVBs), for instance, instead of concentric rings, spiral patterns capable of revealing both the magnitude (number of distinct spirals composing the pattern) and signal (orientation of the turns) were observed [41]. We studied SSPM of OVBs with different topological charges (m=0,±1, ±2, ±3, ±4) after the interaction with liquid suspensions of 2D LTMDs (semiconductor MoS_2_, metallic NbS_2_, and semi-metallic WTe_2_) [42]. Spectral regions of high (532 nm) and low linear absorption (790 nm) were employed again. The main results of refs. [26,42] indicated that thermal effects play a major role and cannot be neglected, as discussed in Section 3.4. Therefore, care must be taken when inferring the optical properties of materials based on SSPM experiments, since the main role of flakes may only be to generate thermal nonlinearities due to the absorbance of the samples.

#### 2.2.4. Z-Scan

**Figure 5:**
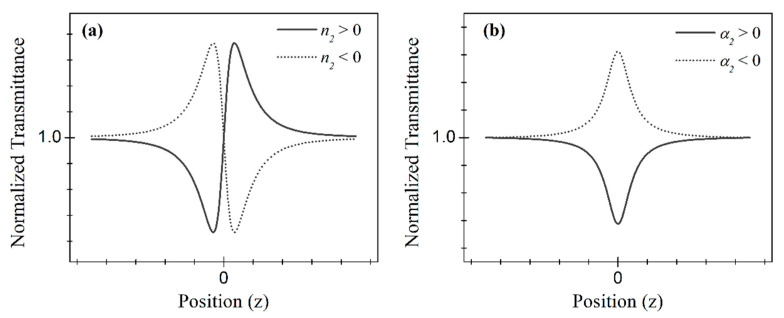
Typical normalized transmittance curves from the Z-scan technique for (**a**) closed and (**b**) open aperture configurations.

The Z-scan method is certainly the most used technique to characterize the third-order response of optical materials, from bulk to nanoscale. It relies on the analysis of the wavefront phase distortion of a beam upon propagation through an NL medium in an excitation intensity regime whereby optical nonlinearity takes place. Experimentally, as shown in Figure 4d, the Z-scan measurement is carried out by inserting a sample in the optical path of a focused beam and translating the sample along its axis through the focal region. When wavefront distortion arises from self-focusing, which occurs when $n_{2}>0$, or defocusing for $n_{2}<0$, the beam intensity detected through a small aperture (compared to the beam cross-section) at the far-field changes with the sample position. Measuring the transmitted intensity through the aperture versus the sample position then allows the determination of the material’s NL refractive index. Without an aperture in front of the detector, such that the whole beam is detected, the material’s NLA coefficient is obtained. The scheme in Figure 4d already shows how these two measurements can be made simultaneously, and the second detector in each arm provides a normalization to the intensity fluctuation of the optical source, which can be a continuous wave or pulsed with a duration from nanosecond to femtosecond, therefore leading to different NLO processes. The typical signatures of the Z-scan directly give the sign of the NLR or, in the case of NLA, if it leads to saturated or multiphoton absorption, as shown in Figure 5.

Quantitatively, it has been shown in the pioneering work of Sheik-Bahae [39] that the peak-to-valley transmittance variation, $\Delta T_{pv}$, at a given wavelength, λ, and for a sample of length, L, in the absence of NLA, is given by:

(4) $$\Delta T_{pv}=0.406\;{\left(1-S\right)}^{0.25}\left(\frac{2\pi L}{\lambda }\right)\Delta n_{0},\;$$

where $\Delta n_{0}=n_{2}I_{0}\;(I_{0}$ being the irradiance at the focus) is the refractive index change at the center of the focus when the third-order nonlinearity is dominant. The above equation is valid for the so-called ‘thin’ samples, and further details can be found in refs. [12,39].

#### 2.2.5. Photoacoustic Z-Scan (PA Z-Scan)

The photoacoustic Z-scan (PA Z-scan) can be seen as an extension of the optical Z-scan, which detects the generation of acoustic (instead of optical) waves, as an optical pulse is absorbed by the sample in the nonlinear regime and is converted into sound waves, as first introduced by [43]. The method relies solely on the material’s absorption, and one of the advantages of its employment with suspensions of scattering materials is that the influence of linear or nonlinear scattering is neglected; it can work with opaque samples and be used with a wide range of excitation wavelengths without the need to change the detector since the detection is in the acoustic regime. An advanced implementation of this technique, termed OPA Z-scan, combines an optical Z-scan and PA Z-scan to obtain a better understanding of the NLA in optical materials [44]. The experimental setup for the OPA Z-scan is shown in Figure 4e.

#### 2.2.6. Optical Kerr Gate (OKG)

The optical Kerr gate (OKG) is a technique that explores the optical Kerr effect to measure the time response and the modulus of the third-order nonlinearity of the materials [12], relying on the polarization rotation of a probe (weak intensity) beam induced by a pump (strong intensity) beam. Figure 4f shows a schematic representation of the experimental setup. In the homodyne regime, i.e., when only the nonlinear birefringence is responsible for the transmitted light after the analyzer, the OKG signal is proportional to $\sin^{2}\left(\frac{\Delta \phi_{NL}}{2}\right)$, which carries direct information about the modulus of $n_{2}$ through the nonlinear phase acquired by the probe, $\Delta \phi_{NL}$ [45]. Conversely, in the heterodyne regime, even in the absence of the pump, a portion of the probe leaks through the analyzer either by linear birefringence, depolarization, or limited extinction of the PBS, and needs to be taken into account [46]. A common treatment is to retrieve information about |$n_{2}|$ by comparing the OKG signal of the investigated material to the signal of a known reference. For this purpose, carbon disulfide (CS_2_) is usually employed to calibrate the system, serving as a reference since its nonlinear properties have long been well characterized and recognized in the literature [47,48]. It is important to emphasize that the properties of the reference material must have been measured in the same temporal, spectral, and polarization regime used in the experiments with the materials of interest since the NLO parameters depend on these attributes, as discussed in ref. [47] for CS_2_.

## 3. Intensity-Dependent Nonlinear Optical Response of 2D LTMDs

### 3.1. Second-Order NLO

**Figure 6:**
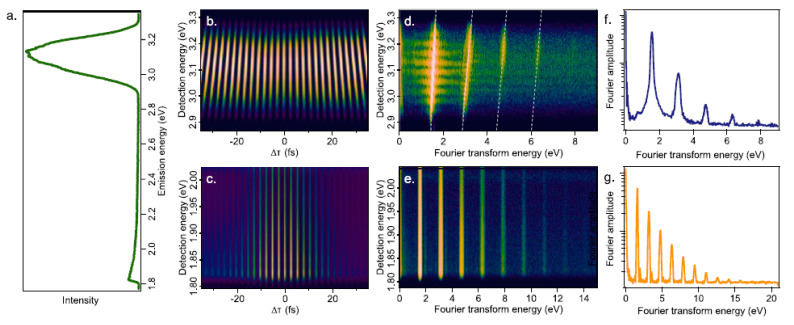
Experimental NLO responses of MoS_2_ prepared via redox-exfoliation analyzed via FT-NLO spectroscopy. (**a**) Nonlinear emission spectrum of MoS_2_ excited at 800 nm (corresponding to 1.55 eV in energy). (**b**) Spectrally resolved spectrogram centered at 3.1 eV (second harmonic of the fundamental excitation). (**c**) Spectrally resolved spectrogram centered at 1.9 eV. (**d**) Correlation map obtained by FT along Δτ of data in graph (**b**). (**e**) Correlation map obtained by FT along Δτ of data in graph (**c**). (**f**) Data in graph (**d**) integrated over energy between 2.9 eV and 3.3 eV. (**g**) Data in graph (**e**) integrated over energy between 1.8 eV and 2.0 eV (Reproduced from ref. [23] with permission).

For materials with a non-centrosymmetric structure, even-order NLO responses may be observed after light–matter interaction. For centrosymmetric materials, the observation of such effects is possible by exploring the interaction of light–matter in regions where symmetry is broken, as in surfaces, for example. The latter is usually the case when exploring even-order NLO effects in LTMDs. Second-harmonic generation (SHG), in particular, is a coherent effect widely explored in thin films of different LTMDs [16,49] (and refs. therein), but is still limited to distinct structures such as liquid suspensions. In recent work, Steves et al. used the FT-NLO technique to investigate the SHG of suspended MoS_2_ prepared via the redox exfoliation method by employing an 800 nm, 20 fs, Ti:sapphire oscillator [23]. Figure 6a is the nonlinear emission spectra of the material, showing a dominant peak at 3.1 eV, corresponding to SHG, and a broad peak around 1.9 eV, which was attributed to multiphoton photoluminescence (MPPL). Figure 6b,c are the spectrally resolved interferograms composed of several emission spectra as a function of the temporal separation between the pulses. The Fourier transform of Figure 6b leads to Figure 6d, where the inclined lines reveal the coherent character of the signal centered at 3.1 eV since harmonic generator signals present a correlation between the excitation and detected frequencies [23]. Figure 6e is the FT of Figure 6c, where the vertical lines indicate the presence of noncoherent signals as expected for multiphoton photoluminescence (MPPL).

**Figure 7:**
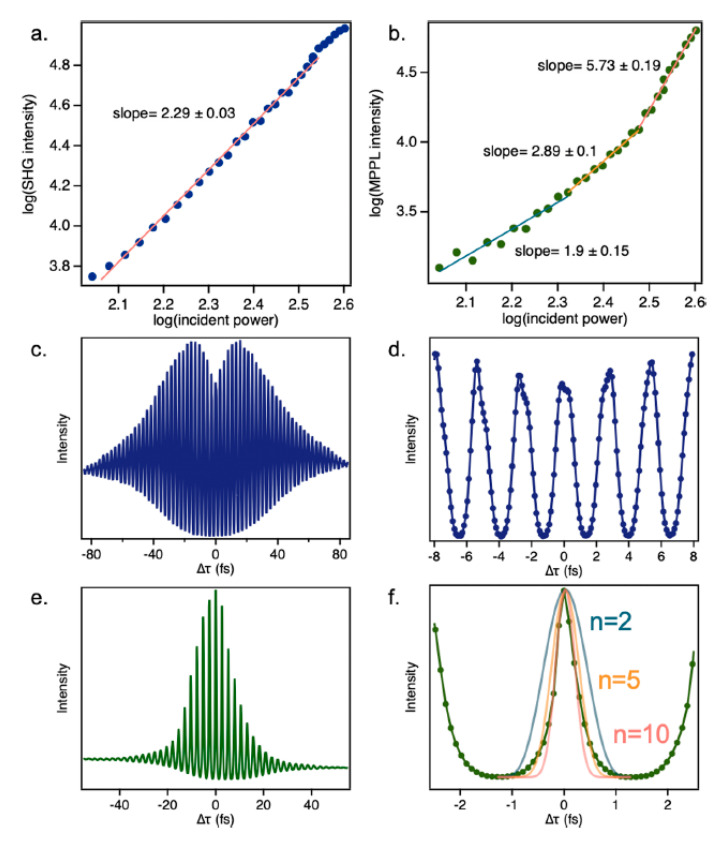
NLO intensity as a function of excitation intensity for (**a**) SHG signal; (**b**) MPPL signal. Interferometric correlation of SHG signal in a (**c**) wider and (**d**) narrower time-span. Interferometric correlation of MPPL signal in a (**e**) wider and (**f**) narrower time-span.

The peaks at different harmonics of the fundamental frequency (ω) in Figure 6d,e indicate their presence in the NLO response. This is clearer in Figure 7f,g, which correspond to Figure 6d,e integrated over the energy between 2.9 eV–3.3 eV and 1.8 eV–2.0 eV, respectively, being equivalent to the FT of the interferometric correlation in the time domain. The presence of 1ω and 2ω in Figure 6d,f is expected for the SHG signal, but the presence of higher-order terms is attributed to the presence of POMs, contributing to the NLO response. As for the MPPL, the FT-NLO technique resolved up to a 10th-order NLO effect (10ω), as seen in Figure 6g. These high-order effects are not disclosed by the usual power dependence of the NLO intensity ($I_{NLO}$) as a function of the excitation intensity ($I_{exc}$), i.e., $I_{NLO}\propto I_{exc}^{n}$ (Figure 7a,b, where n is the order of the effect), an indication of how powerful the FT-NLO technique can be.

The interferometric correlation of the SHG signal shows a decrease around Δτ=0 (Figure 7c), which indicates a saturation of SHG associated with the presence of 3ω and 4ω in Figure 6d,f. It also shows an asymmetry in the interferometric pattern (Figure 7d). None of these features are observed in the interferometric correlation of the signal associated with MPPL (Figure 7e,f), which are consistent with the presence of several distinct orders up to the 10th. As discussed in Section 2.1, the presence of POMs as a byproduct is inherent to the fabrication of redox-exfoliated samples, which may influence the NLO responses. For LTMDs fabricated by other methods that are free of POMs, no saturation of the SHG was reported for the CdSe films investigated by Steves et al. [16,23] (and refs. therein). Conversely, a saturation of SHG was observed for redox-exfoliated WS_2_ and films of CdSe after the addition of POMs, which confirms the contribution of POMs in the NLO response. The observation of MPPL with contributions up to the tenth order is also related to the presence of POMs in the samples. These conclusions are supported by theoretical modeling of the interferometric signal based on the density matrix approach, as discussed in ref. [23].

**Figure 8:**
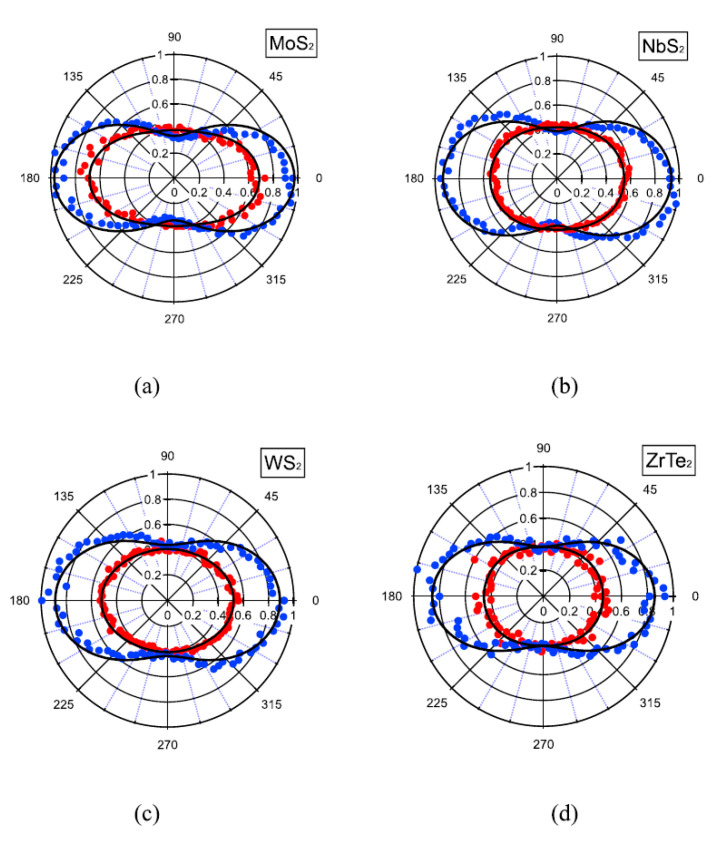
Polar plot of HRS intensity for (**a**) MoS_2_, (**b**) NbS_2_, (**c**) WS_2_, and (**d**) ZrTe_2_ for V polarized (blue circles) and H polarized (red circles) harmonic intensity. The black lines were fitted using Equation (5) (From ref. [24] with permission).

Another technique to explore second-order NLO response is the HRS, as described in Section 2.2.2. In ref. [24], we employed a polarized-resolved HRS by using an 800 nm, 140 fs, and 80 MHz excitation source to characterize four distinct liquid suspensions of LTMDs, namely, semiconducting MoS_2_ and WS_2_, metallic NbS_2_, and semi-metallic ZrTe_2_. Due to the nanoscale size of the flakes, a direct measurement of the hyperpolarizabilities was possible in our experiments. Acetonitrile, which is the solvent of the suspensions, was used as the external reference. Figure 8 shows the HRS intensity as a function of the angle for vertically (blue) and horizontally (red) polarized light (with respect to the laboratory frame). As can be seen, the HRS intensity patterns associated with the vertical polarization present two lobes for all materials, with a deviation that does not exceed 20%. As for the horizontally polarized light, flattened circular plots appeared for all materials. The resulting HRS intensity as a function of the polarization state can be written as [24,25] (and refs. therein):

(5) $$I_{HRS}^{\Gamma }=a^{\Gamma }\cos^{4}\gamma +b^{\Gamma }\cos^{2}\gamma \sin^{2}\gamma +c^{\Gamma }\sin^{4}\gamma ,$$

where $I_{HRS}^{\Gamma }$ is the HRS intensity, Γ corresponds to the H or V polarization state, and the coefficients $a^{\Gamma },\;b^{\Gamma },\;c^{\Gamma }$, which are obtained from the theoretical fits of the experimental data (black lines in Figure 8), determine the depolarization ($D^{V}$), the vertical ($\zeta^{V})$, and horizontal ($\zeta^{H})$ retardation coefficients. The values for all investigated materials are presented in Table 2.

The expected depolarization ratio for a flat octupolar symmetry nonlinearity is two-thirds, which is different from the results obtained in ref. [24] (Table 2). A possible explanation is that the POMs adsorbed in the surfaces of the nanoflakes contribute to the NLO response, as in the case of the FT-NLO study previously discussed [23]. The edges of the nanoflakes or an octupolar symmetry breaking due to the flexibility of the nanoflakes in suspension may also contribute to the deviation of $D^{V}$ in comparison with the value expected to perfect geometries. As for the retardation coefficients, although they do not vanish, they indicate weak retardation for all materials, which may be explained by the strong volume origin of the nonlinearity due to the non-centrosymmetry of the crystal lattice. A model based on a distribution of local non-polarizable nonlinear dipoles was introduced to support the findings [24].

In previous work, we focused our attention on the HRS of ZrTe_2_ suspended in acetonitrile in the nanosecond regime [25]. An Nd:YAG laser operating at 1064 nm, 7 nm, and 10 Hz was employed as the excitation source, and *para*-nitroaniline (*p*-NA) was used as the reference. The orientation-averaged first-hyperpolarizability measured was $\beta \left(2\omega \right)=\left(7.0\pm 0.3\right)\times 10^{-24}$ esu per ZrTe_2_ nanoflake, the largest reported to date. An investigation of the HRS intensity versus concentration of ZrTe_2_ (in the range between $0.5\times 10^{10}$ and $4.9\times 10^{10}$ particles per cm^3^) revealed a linear behavior, indicating that the second-harmonic signal is not due to aggregates, but individual nanoflakes. Polarization-resolved experiments were also performed to identify the origin of the second-order response, indicating an electric dipole origin. The polar plots were similar to the ones presented in Figure 8, from where the coefficients $a^{\Gamma },\;b^{\Gamma },\;c^{\Gamma }$ were obtained after theoretical fit by using Equation (5). Table 3 shows the obtained values together with the depolarization ratio, $\rho^{\Gamma }$, and the multipolarity, $\zeta^{\Gamma }={1-(a}^{\Gamma }+c^{\Gamma }+b^{\Gamma })$, for both polarizations employed (vertical and horizontal). The dipole nature of the NLO response is supported by the relation presented by the coefficients, i.e., $2a^{H}\approx 2c^{H}\approx b^{H}$ and $c^{H}\approx c^{V}$. For a complete discussion, see ref. [25].

### 3.2. NLR and NLA from Z-Scan and Photoacoustics Z-Scan

The third-order response of LTMD nanomaterials can be studied in different spectro-temporal regimes, and this sub-section summarizes the results of such responses using excitation sources in the femtosecond (100–150 fs) or nanosecond (5–10 ns) regime at different excitation wavelengths, which could fall in a high absorptive regime of semiconducting (above optical bandgap) or semimetallic/metallic 2D LTMDs, or a low absorptive regime (below bandgap for semiconducting 2D LTMDs).

**Figure 9:**
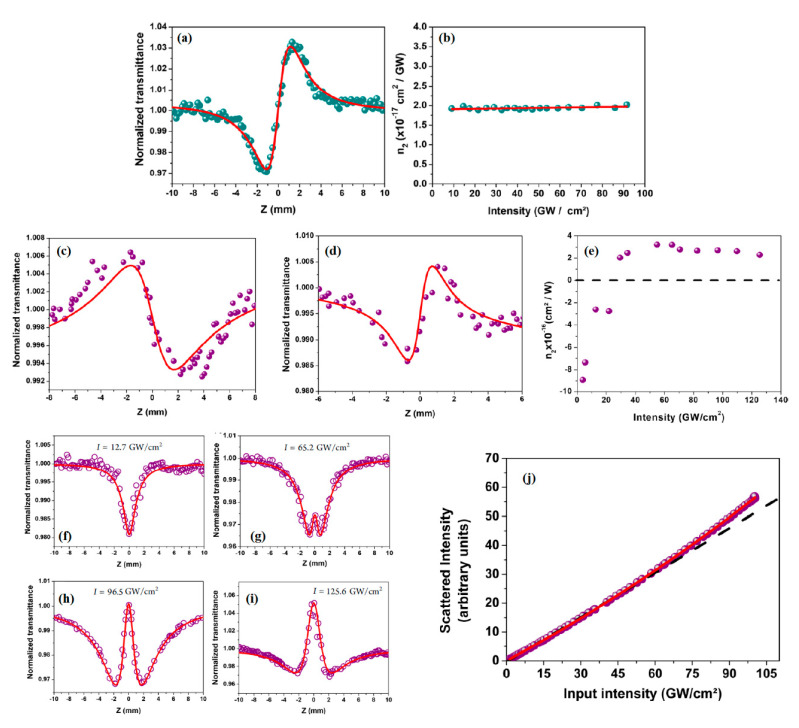
(**a**) Closed-aperture Z-scan curve for ACN at I = 70 GW/cm^2^ (*F* = 44 mJ/cm^2^); (**b**) intensity dependence of $n_{2}$; and the continuous lines in (**a**,**b**) are theoretical fits, see text. Closed-aperture measurements for NbS_2_ (**c**) below the critical intensity of ∼22 GW/cm^2^ (*F* = 34.9 mJ/cm^2^), (**d**) above the critical intensity, and (**e**) effective $n_{2}\times I$ plot, showing the critical intensity whereby the sign of the NLR changes, $I_{C}$ ∼ 25 GW/cm^2^. NLA measurements for sample NbS_2_ for (**f**) I = 12.7 GW/cm^2^ (*F* = 8 mJ/cm^2^), (**g**) I = 65.2 GW/cm^2^ (*F* = 41 mJ/cm^2^), (**h**) I = 96.5. GW/cm^2^ (*F* = 60.7 mJ/cm^2^), and (**i**) 125.6 GW/cm^2^ (*F* = 79.1 mJ/cm^2^). (**j**) Scattered light intensity behavior versus the input laser intensity in the NbS_2_ suspension (Adapted from ref. [22], with permission).

Exploiting the Z-scan method described in Section 2.2 with an optical source from a regenerative amplifier operating at 100 fs, 1 kHz, and 800 nm, we studied the NLR and NLA of metallic NbS_2_ [22], semiconducting MoS_2_ and WS_2_ [27], and semimetallic ZrTe_2_ [28]. Metallic NbS_2_ was the most interesting 2D LTMD studied among the four materials cited above. It was the only one to present both NLR and NLA in the intensity range employed and which would not cause damage to the sample. The main results for the NLR and NLA in the NbS_2_ suspension (in ACN) from ref. [22] are shown in Figure 9. It should be clearly emphasized that all the measured nonlinear coefficients are for the colloidal composite, and not for a single nanoflake.

Initially, as a good practice and control experiment, the solvent (ACN in this case) was characterized and its NLR was measured. It can be directly seen from Figure 9a that it shows a self-focusing, positive NLR with a measured value of $n_{2}$(ACN) = 1.9 × 10^−17^ cm^2^/W. The $n_{2}\times I$ curve shown in Figure 9b was a constant, therefore indicating that third-order NLR was the dominant mechanism (see ref. [12] and refs. therein for basic discussions).

Figure 9c–e for metallic NbS_2_ show quite different and novel behavior for the NLR. Below a critical intensity of ∼22 GW/cm^2^, a negative (self-defocusing) NLR response dominates, which shows an inversion to positive (self-focusing) above this critical intensity. This is notoriously seen in the Z-scan profiles of Figure 9c,d, which correspond to the closed-aperture signal of the NbS_2_ suspension normalized by its open-aperture signal, revealing the focusing/defocusing character presented by this material for intensities above/below the critical intensity of ∼22 GW/cm^2^. In Figure 9c, the intensity is well below the critical intensity, to the point of reverting the closed-aperture pattern if compared with the signal of the pure solvent, which presented a positive n_2_ for the entire range of intensities employed. Figure 9d shows the closed-aperture signal for an optical intensity close to the critical, where the positive character of the nonlinearity can already be seen. It is important to point out that the measured n_2_ values from the Z-scan traces of NbS_2_ in suspension are a resulting nonlinear refractive index, which contains the contribution of the solvent. The influence of the solvent at all intensities may be considered to obtain the contribution of the flakes for the nonlinearity of the sample. The dependence of n_2_ on the optical intensity disregarding the nonlinearity of the solvent (based on the formalism of ref. [50]) is presented in Figure 9e. For intensities above the critical value, the measured n_2_ is (3.0 ± 0.2) × 10^−16^ cm^2^/W. The NLR coefficient values were calculated using Equation (4), as shown in the fits of the solid curves, and further details can be found in ref. [22]. The NLA coefficient was also observed in the NbS_2_ suspension, as shown in Figure 9f–i. The NLA profile varies as a function of intensity, changing from two-photon absorption (TPA) to two-photon saturated absorption (TPSA) at an intensity of *I* = 65.2 GW/cm^2^. This behavior is attributed to the change in the NLR response after saturation is reached, and the nonlinear refractive index changes accordingly via a nonlinear Kramers−Kronig relation. The solid curves in the NLA results are theoretical fits from Equation (6) below:

(6) αI=α0+α2I1+I2IS2+ηI,

where *α*_0_ is the linear absorption coefficient, *α*_2_ is the TPA coefficient, *I_S_* is the TPA saturation intensity, and *η* is the nonlinear scattering coefficient.

The nonlinear scattering term was introduced in Equation (6) because it has been observed from the experimental results shown in Figure 9j, which shows the scattering intensity as a function of input intensity—after a given intensity (around 75 GW/cm^2^) the scattering deviated from linear. To properly fit the results for the NLA, it was then necessary to introduce the NLS (nonlinear scattering) term.

The physical origin of the nonlinearity in NbS_2_ was qualitatively understood with the support of the calculated electronic band structure of the monolayer NbS_2_ 2H phase, as reported in (ref. [22]). Taking into account the conservation of angular momentum, for one- and two-photon transitions, and considering that the material is doped with electrons, it was shown that electronic transitions for TPA in a van Hove singularity at the K point could also occur. It is important to notice that for this van Hove singularity, there is a peak in the density of states that can increase the optical absorption. It was concluded, from the DFT calculation, that both one-photon absorption and TPA were allowed and that the two-photon process could involve a van Hove singularity, which would greatly enhance the NLA. Furthermore, doping could be invoked to explain, via Pauli blocking, the negligible absorption at 800 nm (1.55 eV) (see the absorption spectra, Figure 3c). The negative $n_{2}$ sign for intensities below the critical intensity to induce the NLR sign change is consistent with the theoretical insights, making the NbS_2_ nanoflakes an interesting material for NLO studies and potential applications, whereby control of the optical nonlinearities could be performed. We have also studied semi-metallic ZrTe_2_ under the same experimental conditions, which showed only NLR, whose discussion we defer to ref. [28].

**Figure 10:**
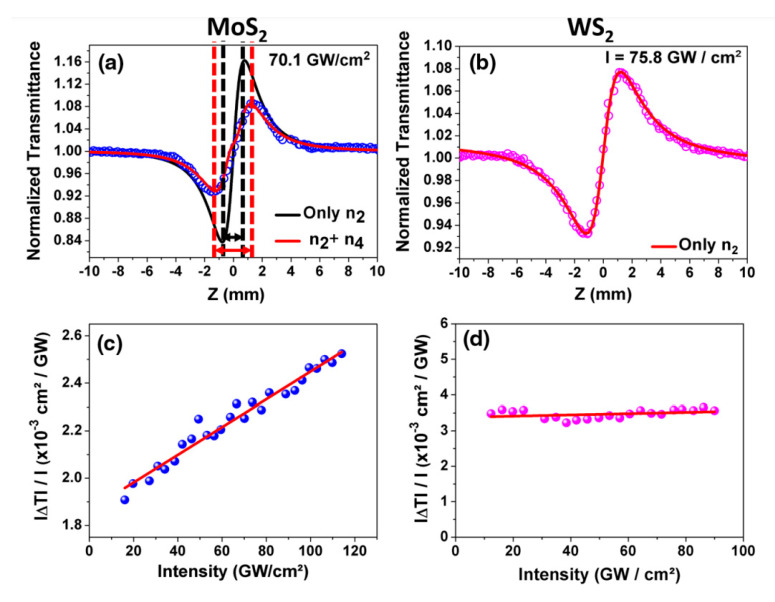
(**a**) Closed aperture Z-scan profile for MoS_2_; the double arrow indicates the peak-to-valley Z-distance, showing that it is larger for the fifth-order curve (red arrow) than for the third-order one (black arrow). (**b**) The same for WS_2_. In both cases, the dots are the experimental results, and the solid lines are the theoretical outcomes; $\left(\left|\Delta T\right|/I\right)$ dependence on I for (**c**) MoS_2_ and (**d**) WS_2_. Reproduced from ref. [27] with permission.

Semiconducting MoS_2_ and WS_2_ are certainly the most studied 2D LMTDs regarding basic understanding and applications, as already pointed out. Figure 10 reproduces the results of our work reported in ref. [27] for the NLO behavior of WS_2_ and MoS_2_ under the same experimental conditions employed to study NbS_2_ and ZrTe_2_. The NLO response for WS_2_ (Figure 10b) shows a positive NLR and a constant intensity dependence in the intensity range studied (shown in Figure 10d). This points to a dominant third-order response, but no NLA was detected for the intensity range studied. Conversely, MoS_2_ showed a distinct behavior, with a clear manifestation of higher-order contribution which we attribute to the fifth-order nonlinearity. It can be seen in the Z-scan signature of Figure 10a) that the different peak-to-valley distance in the z-direction (highlighted by the arrows) is a signature of higher-order contribution to the NLO response of the medium (see ref. [27] and refs. therein). The fit (heavy line) agrees well with the experimental data (dots) only when the fifth-order term is included. Furthermore, in Figure 10c, the intensity dependence of the nonlinear transmittance is linear, again indicating a higher-order nonlinear contribution. Therefore, the measured NLR is in fact an effective value including a contribution of third and fifth-order susceptibilities since the Z-scan methods do not discriminate between each order. Such discrimination can be obtained by employing an angularly resolved four-wave mixing setup, as reported in [51,52].

Using the appropriate equations for $n_{2}$ and $n_{4}$ (see ref. [27]), the effective NLR indices for the MoS_2_ nanoflakes suspension were measured to be $n_{2,eff}=$ (4.8 ± 0.5) × 10^−16^ cm^2^/W and $n_{4,eff}=-$(7.6 ± 0.5) × 10^−26^ cm^4^/W^2^. For WS_2_, the obtained third-order refractive index was $n_{2,eff}=$ (3.4 ± 0.5) × 10^−16^ cm^2^/W. Although the values of the effective NLR coefficients are quite close for MoS_2_ and WS_2_, the fact that the fifth-order NLR manifests only in the MoS_2_ was explained based on the morphology of the samples. Taking into account the extinction spectra (see Figure 3) and the respective nanoflakes’ concentrations of 70 μg/mL for MoS_2_ and 40 μg/mL for WS_2_, and their respective atomic weights of 160 Da and 248 Da for MoS_2_ and WS_2_, respectively, the MoS_2_ colloid had 2.7 times more MoS_2_ nanoflakes than in the WS_2_ case. Inasmuch, as the nanoflakes’ average lateral extensions are 84 nm for MoS_2_ and 325 nm for WS_2_, the ratio between the nanoflakes’ areas is 15. Considering the higher concentration of MoS_2_, it leads to ~40 times more nanoflakes in the suspension. As a result, for the same intensity range, the fifth-order response is manifested in the MoS_2_ and not in the WS_2_.

As observed in the study of NbS_2_ using the Z-scan technique, the effect of nonlinear scattering may affect the nonlinear loss. One way to measure the NLA effect without the influence of nonlinear scattering is to employ the photoacoustic Z-scan, as described in Section 2.2.5, since it relies only on the absorption of the incident light. The results shown in Figure 12 reveal the NLA signatures obtained simultaneously in the all-optical conventional Z-scan and using an acoustic detector, as reported in ref. [29], for MoS_2_, NbS_2_, and ZrTe_2_, whose experimental setup is shown in Figure 4e. The light source employed was an optical parametric oscillator operating at 532 nm, 10 Hz, with pulses of 5 ns duration.

**Figure 11:**
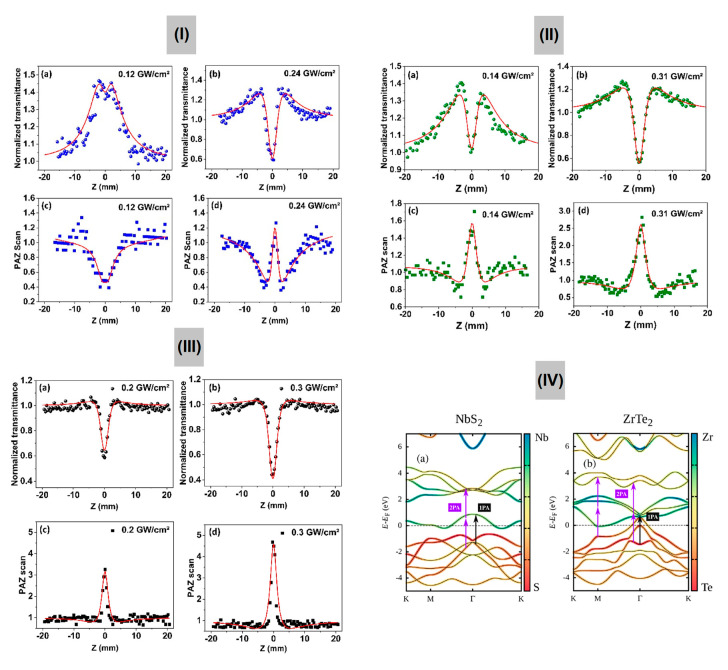
**Frame I:** OZ-scan and PA Z-scan results for MoS_2_. (**a**,**b**) Open aperture optical Z-scan signatures for different intensities. (**c**,**d**) Respective photoacoustic Z-scan signatures. **Frame II:** OPA Z-scan results for NbS_2_. (**a**,**b**) Open Z-scan signatures for different intensities. (**c**,**d**) Respective photoacoustic Z-scan signatures. **Frame III:** OZ-scan and PA Z-scan signatures for ZrTe2. (**a**,**b**) Open Z-scan signatures for different intensities. (**c**,**d**) Respective photoacoustic Z-scan signatures. **Frame IV:** Electronic band structure and optical vertical transitions for one-photon absorption and two-photon absorption at 532 nm: (**a**) NbS_2_ monolayer; (**b**) ZrTe_2_ monolayer (Adapted from ref. [29], with permission).

For an optical intensity of 0.12 GW/cm^2^, the normalized transmission curve from OZ-scan for MoS_2_ revealed an increase in the transmittance near the focus, as shown in Figure 11(Ia). This indicates a predominance of saturable absorption (SA) over other NLA mechanisms, and a small dip around the focus points out a change in the nonlinear behavior for higher intensities. Indeed, for I= 0.24 GW/cm^2^, a clear valley inside the peak at the focus (z = 0) indicates that the reverse saturable absorption (RSA) is prominent (Figure 11(Ib)). RSA may have different origins, such as nonlinear scattering (NLS), nonlinear multiphoton absorption, or intensity-dependent photogenerated absorption, so a complementary technique to disclose the physical mechanisms responsible for RSA is important. For this purpose, we employed the PA Z-scan that is insensitive to scattering processes, leading to very distinct signals when NLA or NLS is the dominant effect in the nonlinear losses. For MoS_2_, PA Z-scan resulted in experimental curves that reassemble a mirror image of the OZ-scan (Figure 11(Ic,d)), an indication that NLA is dominant. This conclusion is possible because the photoacoustic (PA) signal is a consequence of acoustic waves generated due to thermal contraction/dilatation associated with the heating/cooling of the samples. In this scenario, a more prominent NLA, which leads to a larger drop in the OZ-scan curves, also leads to acoustic waves of larger amplitude and, therefore, a rise in the PA Z-scan signal. Conversely, when there is a saturation in the absorption of the sample, the transmittance of the OZ-scan rises, as in Figure 11(Ia), while the PA signal decreases (Figure 11(Ic)), explaining why the PAZ-scan curves appear to be a mirror image of the OZ-scan when the NLA is dominant in the nonlinear losses.

**Figure 12:**
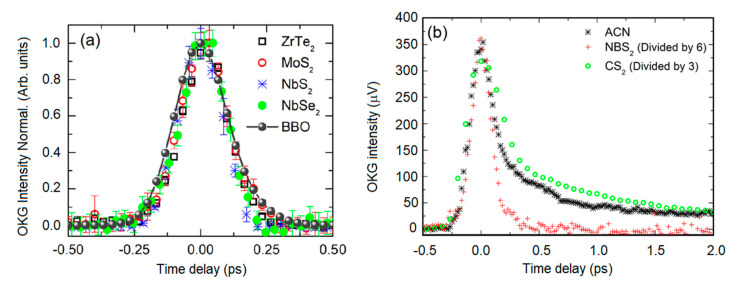
(**a**) Normalized OKG signal as a function of the delay between pump and probe for (**a**) metallic NbS_2_, metallic NbSe_2_, semiconducting MoS_2_, and semimetallic ZrTe_2_. Gray dots connected by a solid line indicate the intensity correlation of pump and probe pulses by using a BBO crystal for SHG. (**b**) Acetonitrile (ACN), metallic NbS_2_, and CS_2_ (Adapted from ref. [30] with permission).

Figure 11(IIa,b) show the OZ-scan results for NbS_2_, revealing an overall behavior similar to MoS_2_, i.e., a SA character followed by an RSA, but the threshold for the observation of RSA in NbS_2_ is lower since a bigger dip is observed for similar optical intensities, as notice when comparing Figure 11(Ia) to Figure 12(IIa). As for semimetallic ZrTe_2_, Figure 11(IIIa,b) reveal that, for the range of intensities employed, RSA is already dominant, being attributed by the authors to the lower linear absorption coefficient, α_0_, in comparison to that of MoS_2_ and NbS_2_ [29]. The band structure of the materials (Figure 11IV) is also considered in the analysis presented in ref. [29].

In Table 4, we summarize the nonlinear attenuation coefficients (β) for MoS_2_, NbS_2_, and ZrTe_2_ obtained from the OZ-scan and PA Z-scan experiments at 532 nm, 10 Hz, and optical pulses of 5 ns. For OZ-scan, we employed a simple model that considers SA (left term) and RSA as a consequence of third-order nonlinear processes (right term) given by:

(7) $$\alpha \left(I\right)=\frac{\alpha_{0}}{1+\frac{I(z)}{I_{S}}}+\beta I,\;$$

where $\alpha_{0}$ is the linear absorption coefficient, $I_{S}$ is the saturation intensity, and β is the nonlinear attenuation coefficient, which can include contributions due to NLA and NLS, and I(z) is the optical intensity. As for the PA Z-scan, since the amplitude of the acoustic signal (P) is proportional to the absorption coefficient in such a way that $P\left(z\right)=\Gamma \alpha \left(I\right)I(z)$, where Γ is the Grüneisen coefficient, we considered the following expression:

(8) $$P_{N}\left(z\right)=\frac{I_{S}}{I_{S}+I\left(z\right)}+\frac{\beta }{\alpha_{0}}I\left(z\right).$$

Knowing that the laser intensity within the sample evolves along the z-optical axis according to $dI(z)/dz=-\alpha \left(I\right)I$, we can solve it numerically (by considering Equation (7) for the case of OZ-scan, and Equation (8) for PA Z-scan) to retrieve information about the β of the samples. The red lines in Figure 11 are theoretical fits by using this procedure, which resulted in the values represented in Table 4. Discrepancies between the β from PA Z-scan and OZ-scan are justified since the former, obtained by Equation (8), is only sensitive to NLA, while the latter (Equation (7)) can also have contributions from NLS.

### 3.3. Measurements of Time Response through OKG

We employed the OKG technique to study the third-order optical response of 2D metallic NbS_2_ and NbSe_2_, semiconducting MoS_2_, and semimetallic ZrTe_2_, all suspended in acetonitrile [30]. An ultrafast femtosecond laser (180 fs, 800 nm, 76 MHz) was used to generate both pump and probe beams. All investigated materials showed a fast response that followed the temporal width of the optical pulses, Figure 12a, indicating the dominance of electronic nonlinearity ([12] and refs. therein). As for pure ACN, Figure 12b, a slow decay time of 1.66 ps reveals an orientational contribution that is not present in the LTMDs, reinforcing that the 2D flakes enhance the nonlinearity of the samples making the electronic response predominant [46]. The highest NLO response was presented by NbSe_2_ with an $\left|n_{2}\right|=5.3\times 10^{-18}$ m^2^/W, followed by MoS_2_ with $4.8\times 10^{-18}$ m^2^/W, ZrTe_2_ with $2.7\times 10^{-18}$ m^2^/W, and NbS_2_ with $9.3\times 10^{-19}$ m^2^/W. NLA was not observed for any studied material, even at the maximum available intensity of ~100 MW/cm^2^.

As discussed in Section 3.2, we also used the Z-scan technique to measure refractive nonlinearities of MoS_2_ [27], ZrTe_2_ [28], and NbS_2_ [22] from which we obtained $n_{2}=4.5\times 10^{-20}$ m^2^/W, $4.2\times 10^{-20}$ m^2^/W, and $3.0\times 10^{-20}$ m^2^/W, respectively, one to two orders of magnitude lower than the ones measured by OKG. However, the intensities employed in the Z-scan were ~100 GW/cm^2^, three orders of magnitude larger. From the analysis of the Z-scan results, a strong intensity dependence of $n_{2}$ was found, which may explain the difference when compared with the results from OKG. For NbS_2_, for instance, ${|n}_{2}|$ exhibits an asymptotic behavior that increases at low intensities [22], which is consistent with the results from OKG, which employed optical intensities of ~100 MW/cm^2^. These results are summarized in Table 5. The physical mechanism related to the ultrafast time response of NbS_2_, NbSe_2_, MoS_2_, and ZrTe_2_ that considers the nature of the 2D material is discussed in ref. [30].

It is worth noting that the data given in Table 5 (and in Table 6) are dependent on the suspension concentration, as it is an effective value. In each of the related references, the absorbance spectra for the particular sample employed are given, and that takes into account the concentration of that particular sample, making the reported data meaningful.

### 3.4. Thermal Response

**Figure 13:**
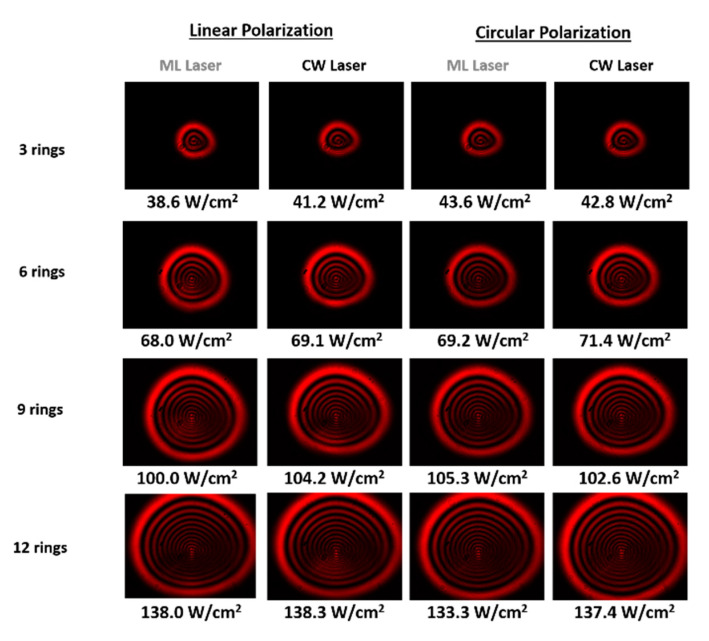
SSPM patterns for Gaussian beam after interaction with the liquid suspension of MoTe_2_ at 790 nm (CW and ML laser, 120 fs, 76 MHz), showing no dependence on either polarization or the temporal regime of excitation. Optical intensities are indicated. Cuvette: 1 cm (Reproduced from ref. [26] with permission).

**Figure 14:**
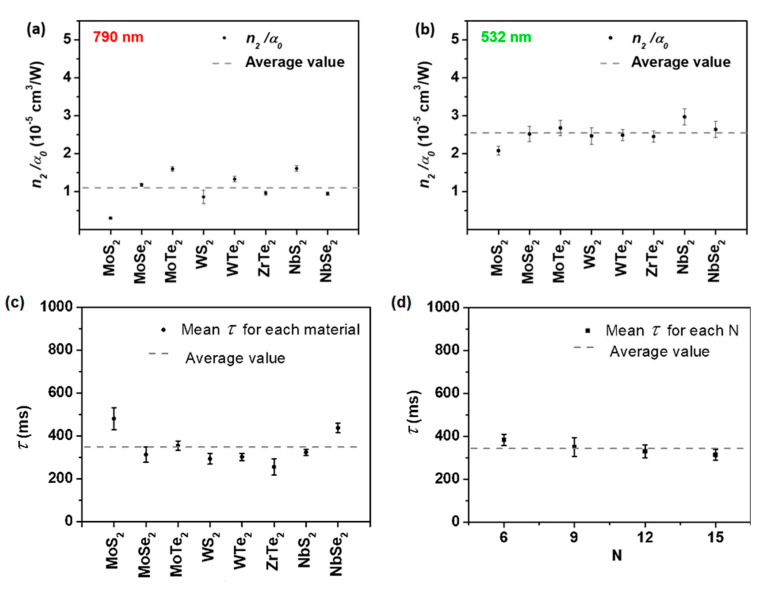
Ratio n_2_/α_0_ for the studied materials at (**a**) 790 nm (CW and ML laser, 120 fs, 76 MHz) and (**b**) 532 nm (CW laser). The average values are 1.10 × 10^−5^ cm^3^/W at 790 nm and 2.54 × 10^−5^ cm^3^/W at 532 nm. The error bar is the standard error of the mean value. (**c**) The mean value of time for ring formation considering 6, 9, 12, and 15 rings for each material separately (dots). (**d**) The mean value of time for ring formation considering all materials for each number of rings (dots). The average value (dashed lines) is 344.9 ms (Reproduced from ref. [26] with permission).

As introduced in Section 2.2.3, we performed experiments with eight distinct LTMDs suspended in acetonitrile, namely, semiconductor MoS_2_, MoSe_2_, MoTe_2_, WS_2_, semimetallic WTe_2_, ZrTe_2_, and metallic NbS_2_, NbSe_2_, to characterize the physical mechanisms responsible for the SSPM [26]. To obtain a wide picture and have an insight into the NLO responses, CW and ML (120 fs, 76 MHz) lasers in the spectral regions of low (790 nm) and high (532 nm) linear absorption of the samples were employed. The spatial self-phase modulation of Gaussian beams, as used in the experiments of ref. [26], gives rise to a nonlinear radial phase that follows the Gaussian intensity profile. This phase is responsible for the emergence of concentric rings in the diffraction pattern, shown in Figure 13, from which one can retrieve $n_{2}$ based on the dependence of the number of rings with the optical intensity, the effective length of the sample, and the wavelength of the radiation [40]. At 532 nm (CW laser), we obtained $n_{2}$ ranging from $4.9\times 10^{-6}$ cm^2^/W to $42.0\times 10^{-6}$ cm^2^/W, while it presented values between $0.9\times 10^{-6}$ cm^2^/W and $10.1\times 10^{-6}$ cm^2^/W at 790 nm (for both CW and ML laser, 120 fs, 76 MHz). More interestingly, we noticed a clear correlation between the nonlinearity of the samples and their linear absorption coefficient ($\alpha_{0}$), an indication that the SSPM is due to a slow thermal nonlinearity. This became evident after analyzing the ratio $\left(n_{2}/\alpha_{0}\right)$ for all materials, which presented very close values with an average of 1.10 × 10^−5^ cm^3^/W at 790 nm and of 2.54 × 10^−5^ cm^3^/W at 532 nm, as illustrated in Figure 14a,b. Therefore, it is possible to conclude that the role of the 2D flakes is in absorbing the radiation to heat the suspension, while a thermal lens is established in the liquid environment according to the thermal properties of the solvent (which is the same for all investigated materials, explaining why the ratio is so close for all studied materials). Therefore, it is not possible to measure intrinsic NLO properties of the 2D materials from SSPM experiments by using CW or ML lasers with high repetition rate, since the thermo-optic coefficient associated with the thermal nonlinearity responsible for the effect would be very similar to any material that presents similar absorbance and thermal properties. The time response of the NLO effect is also in accord with slow thermal nonlinearities, with an average value of approximately 350 ms, showing no dependence either on the number of rings or on the nature of the 2D material, as shown in Figure 14c,d.

**Figure 15:**
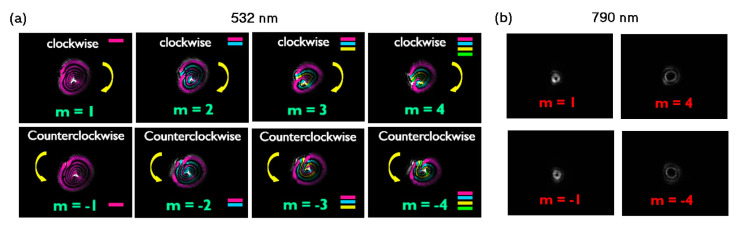
(**a**) SSPM patterns of OVBs with indicated topological charge (m) at 532 nm (CW laser), a spectral region of high absorbance. The optical intensity is 16 W/cm^2^. (**b**) Experimental results at 790 nm (ML laser, 150 fs, 76 MHz), a spectral region of low absorbance. No SSPM was observed. The optical intensity is 17 W/cm^2^. Results for MoS_2_ are suspended in ACN (Reproduced from [53] with permission).

**Figure 16:**
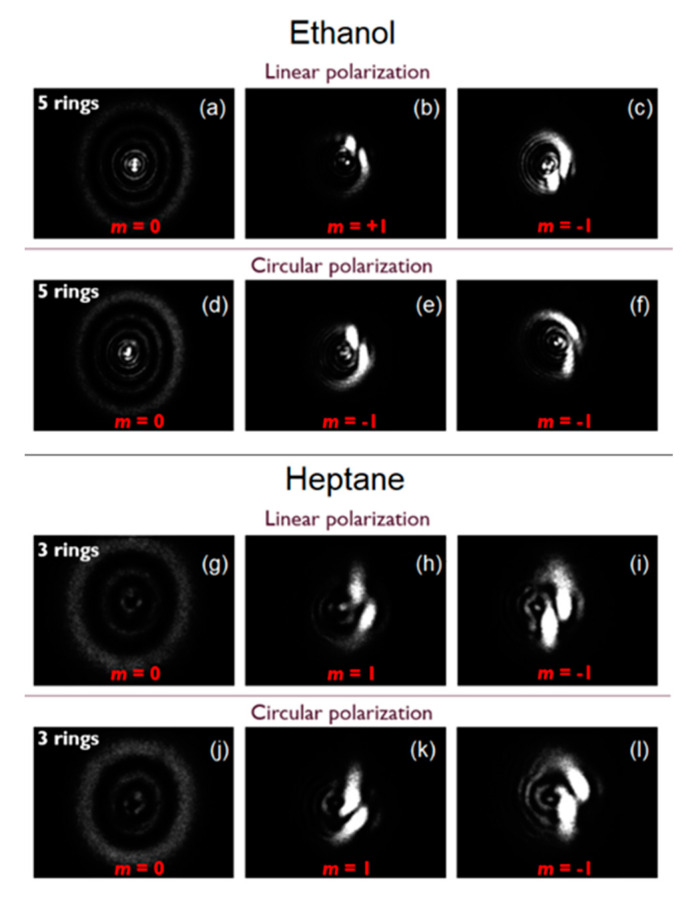
SSPM of OVBs showing spiral features and no dependence on the polarization state for either polar ethanol or nonpolar heptane. The spectral region is 1560 nm (100 fs, 50 MHz). Optical intensities are 10 W/cm^2^. Cuvette: 5 mm for ethanol and 5 cm for heptane (Reproduced from [42]).

In a subsequent study, we explored the SSPM of optical vortex beams (OVBs) with different topological charges (m=0,±1, ±2, ±3, ±4) after interaction with liquid suspensions of 2D LTMDs (semiconductor MoS_2_, metallic NbS_2_, and semi-metallic WTe_2_) [42,53]. Spectral regions of high (532 nm, CW laser) and low linear absorption (790 nm, ML laser, 150 fs, 76 MHz) were employed. As mentioned in the NLO techniques section, for structured light, the nonlinear phase associated with SSPM follows the beam spatial profile, giving rise to distinct diffraction patterns when compared to the SSPM of Gaussian beams. For OVBs, for instance, instead of concentric rings, spiral patterns capable of revealing both the magnitude (number of distinct spirals composing the pattern) and signal (clockwise orientation for positive; counterclockwise for negative) of the topological charge are observed [41]. Figure 15a shows representative patterns for MoS_2_ at 532 nm. In refs. [42,53], after performing SSPM experiments with OVBs, we observed a clear correlation between the nonlinearity of the samples and their absorbance spectra (Figure 15a,b are evidence), which corroborates with ref. [26] for the case of Gaussian beams. It was possible to conclude that a slow thermal nonlinearity is the physical mechanism underlying the NLO response, regardless of the nature of the 2D material employed. In fact, for any material with similar absorbance and thermal properties, similar SSPM results would be achieved. This conclusion was supported by the SSPM of OVBs in pure solvents (without the presence of the 2D flakes), which resulted in similar spiral patterns as long as the experiment was performed in spectral regions of high absorbance, as shown in Figure 16 for ethanol and heptane at 1560 nm. The SSPM of both 2D flakes in suspension and pure solvents, for both OVBs and Gaussian beams, also proved to be independent of the temporal regime of the excitation optical beam employed (CW or ML with high (~MHz) repetition rate) and light polarization, as shown in Figure 13 and Figure 16, another indication that thermal effects play a major role and cannot be neglected in experiments similar to the ones performed in refs. [16,42,53]. Therefore, care must be taken to infer intrinsic electronic properties of materials based on SSPM experiments, especially when CW or ML lasers with a high (~MHz) repetition rate are employed.

Table 6 brings a summary of the measured ${|n}_{2}|$ from the SSPM experiments (thermal nonlinearity), Z-scan, and OKG (electronic nonlinearity) experiments for comparison. Notice that the difference in magnitude is up to 10 orders, reinforcing that a thorough analysis concerning the physical mechanism responsible for the effect is important, otherwise mistaken overestimated electronic properties of the materials could be inferred based on thermal nonlinearities, which bear no relationship.

## 4. Challenges and Opportunities

### 4.1. Fundamentals

This review reported the NLO response of monolayer to a few layers of semiconducting, semimetallic, and metallic 2D-LTMDs suspended in acetonitrile, from which the nonlinear refraction (NLR) index arises from the thermal origin. Therefore, a high value of the order of ~10^−6^–10^−5^ cm^2^/W and ~10 of ms time response was inferred, as well as from electronic origin, whose NLR index was ~10 orders of magnitude smaller, with a response time of ~150 fs limited by the duration of the laser pulse employed. From a fundamental point of view, working with the nanosheets in suspensions gives all the relevant information from the material, but it should be noted that the obtained value of the nonlinear parameters is indeed for the suspension, and takes into account the value of the nonlinear response of the solvent and the nanosheets concentration. In our case, the result for the solvent itself was at least one order of magnitude smaller than for the suspension, in the case of the non-thermal response, whereas in the case of the thermal response, it was negligible when compared to any of the employed colloidal suspensions. Although we emphasized the third-order nonlinear response, some of the reported work is related to second-order nonlinearities, which is an important area where several earlier works have been reported [54,55,56,57].

The studies with liquid suspensions of LTMDs will contribute to a better basic knowledge of 2D materials. When contrasting with conventional materials such as silicon, germanium, and III-V semiconductors, the LTMDs have unique optical, thermal, electrical, and mechanical properties. For instance, the optical and electronic properties of LTMDs can be controlled by exploiting the large surface-to-volume ratios of these materials. Moreover, the surface of LTMDs materials is passivated without dangling bonds. As a result, it becomes easier to combine different structures such as optical waveguides. Currently, the research on LTMDs is focused on the characterization of nonlinear optical properties and the proof-of-principle demonstration of basic devices. However, we foresee that future advances related to the structural modulation of LTMDs will contribute to the fabrication of optoelectronic and nonlinear optical devices with well-reproduced properties.

Further fundamental studies can give a deeper insight using monolayers (or few layers) 2D-LTMDs on substrates, whereby the single (or multi) layer nonlinear response can be directly obtained. This also opens the possibility of employing layers of different materials, therefore leading to heterostructures [2,58], as well as studying the edge effect or polarization response, besides angular alignment. All experimental variations may require novel theoretical approaches, which can lead to potentially new photonic devices.

### 4.2. Applications

2D-LTMDs have already found a myriad of applications, which includes their uses in solar cells, energy storage and optoelectronic devices, and sensors [2,5,6]. 2D-LTMDs have also been pursued as nanomaterials for optical sources, including quantum dots [59] or as scatterers for random lasers [60]. Other technological advances in rolling 2D-LTMDs nanosheets have been reported [61], which followed on the demonstration of a photodiode based on nanosheets of WSe_2_/MoS_2_ prepared by nanoscroll integration [62]. Among the future applications, sensors for healthcare have been at the forefront of research, as reviewed in ref. [63]. As an important example, reviewed in ref. [64], thermoelectric materials, which can be flexible, user-friendly, and lightweight, have been fabricated with 2D materials. For instance, exfoliating TiS_2_ nanosheets and assembling them with carbon nanocrystal (C60) enabled the development of a new n-type flexible thermoelectric material by simultaneously decreasing the thermal conductivity while increasing the power factor. For large-area printing to make flexible thermoelectric devices, the C60/TiS_2_ solutions are proposed to be used as an ink.

Due to the tunability and ability to capture biomarkers, 2D materials find applications in the biomedical field. However, the toxicity of 2D materials remains a concern, even though an effective reduction in toxicity has been achieved with functionalization.

Another well-known property of certain materials, piezoelectricity, has intrinsic characteristics of TMDs which have recently been explored. In SnS_2_/SnS-based 2D thin films, due to a substantial band offset brought on by the creation of the heterojunction, the piezoelectric response is ~40% higher than that of the pure SnS2 thin film (see [64] and refs therein). The SnS_2_/SnS heterostructure can be potentially used to create an adjustable energy harvesting device, which can also be an attachable, self-powered sensor for tracking heartbeat and other small displacements in the biological system of humans.

In the electronics field, it has been questioned if 2D-LTMDs can replace silicon, or if this is hype [65]. For potential commercialization, full control and reproducibility of materials preparation is of paramount importance as a first step. Regarding applications in optoelectronics, HS (heterostructures)-based 2D materials are rapidly taking off from laboratory to industrial scale. The use of vertical integration monolayers (mechanically isolated) via layer-by-layer transfer process is not a limiting factor on lattice matching, as discussed in [66], since individual layers can be coupled to each other via vdW interaction. Therefore, unparalleled freedom to combine different 2DLMs exploring various exotic functionalities can be performed, which allows us to manipulate features such as layers stacking order, mutual rotation, and the application of external fields. Limitations can arise from interfacial contamination and lack of scalability issues in the 2D transfer process limit. For large-scale applications, wafer-scale samples with electronic grade quality have been a challenge for designing high-performance devices. The existing CVD strategies, which are well-established, need to be revised to obtain precise control over thickness, morphology, and rotational alignment to explore new physics and new devices.

The above examples show the potential for applicability of 2D LTMDs, and the knowledge of the fundamental light–matter interaction in such nanomaterials is definitively worth pursuing.

## Figures and Tables

**Table 1:** Nonlinear polarization and associated wave mixing processes.

Polarization Term		Associated Mixing Process
$P_{0}$	Permanent polarization	None
$P^{\left(1\right)}=\epsilon_{0}\chi^{\left(1\right)}⊗E$	First-order polarization	None
$P^{\left(2\right)}=\epsilon_{0}\chi^{\left(2\right)}⊗EE$	Second-order polarization (considering incident fields of frequencies $\omega_{1}$ and $\omega_{2}$)	$2\omega_{1},\;2\omega_{2},{\;\omega }_{1}\pm \;\omega_{2}$
$P^{\left(3\right)}=\epsilon_{0}\chi^{\left(3\right)}⊗EEE$	Third-order polarization (considering incident fields of frequencies $\omega_{1}$, $\omega_{2}$ and $\omega_{3}$).	$\omega_{1}\pm \;\omega_{2}\pm \omega_{3};3\omega_{n},\;n=1,\;2,\;3;$ $2\omega_{m}\pm \omega_{n\;}(m,\;n=1,\;2,\;3;m\neq n)$

**Table 2:** Depolarization coefficient ($D^{V}$), vertical ($\zeta^{V}$), and horizontal ($\zeta^{H}$) retardation coefficients from polarization plots. Error is about 10% [24].

Sample	$D^{V}$	$\zeta^{V}$	$\zeta^{H}$
MoS_2_	0.35	−0.18	−0.22
WS_2_	0.49	−0.05	−0.06
NbS_2_	0.41	−0.03	0.01
ZrTe_2_	0.45	−0.12	0.04
Acetonitrile	0.28	−0.09	0.02

**Table 3:** Coefficients determined from theoretical fit of the polarization plots of ref. [25] by using Equation (5). $\rho^{\Gamma }=c^{\Gamma }/a^{\Gamma }$ is the depolarization ratio. $\zeta^{\Gamma }={1-(a}^{\Gamma }+c^{\Gamma }+b^{\Gamma })$ is the multipolarity [25].

Coefficient	V Polarization	H Polarization
a	0.89	0.33
b	1.20	0.74
c	0.27	0.25
ρ	0.30	0.75
ζ	0.03	0.21

**Table 4:** Nonlinear attenuation coefficient for MoS_2_, NbS_2_, and ZrTe_2_ obtained from the OZ-scan and PA Z-scan experiments at 532 nm, 10 Hz, and optical pulses of 5 ns [29].

Material	Technique	β (10^−7^ cm/W)	$I_{S}$ (MW/cm^2^)
MoS_2_	OZ-scan	0.53	10
	PA Z-scan	0.20	11.4
NbS_2_	OZ-scan	0.42	8
	PA Z-scan	0.14	10
ZrTe_2_	OZ-scan	0.50	7
	PA Z-scan	0.24	10.3

**Table 5:** NLO parameters for liquid suspensions of NbS_2_, NbSe_2_, MoS_2_, and ZrTe_2_ obtained from OKG (intensities ~100 MW/cm^2^) and Z-scan (intensities ~100 GW/cm^2^) for the similar spectro-temporal regime (800 nm, ~100 fs) (reproduced from ref. [30] with permission).

Material	Technique	$\left|n_{2}\right|$ (m^2^/W)	α_2_ (cm/GW)	Ref.
NbS_2_	OKG	(9.3 ± 0.5) × 10^−19^	Not observed	[30]
NbS_2_	Z-scan	(3.0 ± 0.2) × 10^−20^	2.1 × 10^−1^	[22]
MoS_2_	OKG	(4.8 ± 0.6) × 10^−18^	Not observed	[30]
MoS_2_	Z-scan	(4.5 ± 0.3) × 10^−20^	---	[27]
ZrTe_2_	OKG	(2.7 ± 0.3) × 10^−18^	Not observed	[30]
ZrTe_2_	Z-scan	(4.2 ± 0.3) × 10^−20^	---	[28]
NbSe_2_	OKG	(5.3 ± 0.7) × 10^−18^	Not observed	[30]
CS_2_	Z-scan	(3.1 ± 1.0) × 10^−19^	$1\times 10^{-2}$	[48]

**Table 6:** Range of |$n_{2}$| from SSPM at 790 nm (120 fs, 76 MHz) and from Z-scan experiments at 790 nm in the femtosecond regime. *th* indicates the thermal origin of the nonlinearity.

Material	Wavelength	|n_2,*th*_| (10^−6^ cm^2^/W)	|n_2_| (10^−16^ cm^2^/W)
Semiconducting (MoS_2_, MoSe_2_, MoTe_2_, WS_2_)	790 nm	0.9–10.1 [26]	3.4–4.8 [27,30]
	532 nm	14.6–42.0 [26]	---
Metallic (NbS_2_, NbSe_2_)	790 nm	8.8–4.5 [26]	3.0 [22,30]
	532 nm	34.5–36.2 [26]	---
Semi-metallic (WTe_2_, ZrTe_2_)	790 nm	0.9–5.0 [26]	4.2 [28]
	532 nm	4.9–34.5 [26]	---

## Data Availability

Data is available upon requests to the corresponding author.
